# *Tomato brown rugose fruit virus*: An emerging and rapidly spreading plant RNA virus that threatens tomato production worldwide

**DOI:** 10.1111/mpp.13229

**Published:** 2022-05-22

**Authors:** Shaokang Zhang, Jonathan S. Griffiths, Geneviève Marchand, Mark A. Bernards, Aiming Wang

**Affiliations:** ^1^ London Research and Development Centre Agriculture and Agri‐Food Canada London Ontario Canada; ^2^ Department of Biology The University of Western Ontario London Ontario Canada; ^3^ London Research and Development Centre Agriculture and Agri‐Food Canada Vineland Ontario Canada; ^4^ Harrow Research and Development Centre Agriculture and Agri‐Food Canada Harrow Ontario Canada

**Keywords:** cross protection, emerging virus, RNA virus, seedborne virus, tobamovirus, tomato, *Tomato brown rugose fruit virus*

## Abstract

Tomato brown rugose fruit virus (ToBRFV) is an emerging and rapidly spreading RNA virus that infects tomato and pepper, with tomato as the primary host. The virus causes severe crop losses and threatens tomato production worldwide. ToBRFV was discovered in greenhouse tomato plants grown in Jordan in spring 2015 and its first outbreak was traced back to 2014 in Israel. To date, the virus has been reported in at least 35 countries across four continents in the world. ToBRFV is transmitted mainly via contaminated seeds and mechanical contact (such as through standard horticultural practices). Given the global nature of the seed production and distribution chain, and ToBRFV's seed transmissibility, the extent of its spread is probably more severe than has been disclosed. ToBRFV can break down genetic resistance to tobamoviruses conferred by *R* genes *Tm‐1*, *Tm‐2*, and *Tm‐2* ^*2*^ in tomato and *L* ^*1*^ and *L* ^*2*^ alleles in pepper. Currently, no commercial ToBRFV‐resistant tomato cultivars are available. Integrated pest management‐based measures such as rotation, eradication of infected plants, disinfection of seeds, and chemical treatment of contaminated greenhouses have achieved very limited success. The generation and application of attenuated variants may be a fast and effective approach to protect greenhouse tomato against ToBRFV. Long‐term sustainable control will rely on the development of novel genetic resistance and resistant cultivars, which represents the most effective and environment‐friendly strategy for pathogen control.

**Taxonomy:**

*Tomato brown rugose fruit virus* belongs to the genus *Tobamovirus*, in the family *Virgaviridae*. The genus also includes several economically important viruses such as *Tobacco mosaic virus* and *Tomato mosaic virus*.

**Genome and virion:**

The ToBRFV genome is a single‐stranded, positive‐sense RNA of approximately 6.4 kb, encoding four open reading frames. The viral genomic RNA is encapsidated into virions that are rod‐shaped and about 300 nm long and 18 nm in diameter. Tobamovirus virions are considered extremely stable and can survive in plant debris or on seed surfaces for long periods of time.

**Disease symptoms:**

Leaves, particularly young leaves, of tomato plants infected by ToBRFV exhibit mild to severe mosaic symptoms with dark green bulges, narrowness, and deformation. The peduncles and calyces often become necrotic and fail to produce fruit. Yellow blotches, brown or black spots, and rugose wrinkles appear on tomato fruits. In pepper plants, ToBRFV infection results in puckering and yellow mottling on leaves with stunted growth of young seedlings and small yellow to brown rugose dots and necrotic blotches on fruits.

## Graphical abstract

This pathogen profile summarizes current knowledge about ToBRFV, highlights recent research progress, discusses future research directions, and proposes short‐run and long‐term control strategies.

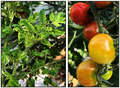

## 1 INTRODUCTION

Tomato (*Solanum lycopersicum*) and pepper (*Capsicum annuum*) are major field and greenhouse vegetable crops grown all over the world (Baenas et al., 2019; Quinet et al., 2019). Like other cultivated crops, tomato and pepper suffer from constant attacks by various pests and pathogens. Viruses are among a few major pathogens that impede tomato and pepper production, and important viruses include, but are not limited to, begomoviruses, tospoviruses, cucumoviruses, potyviruses, and tobamoviruses. Infection by these viruses reduces crop yield and deteriorates fruit quality and marketability, causing substantial economic losses (Hanssen & Lapidot, 2012; Jones & Naidu, 2019; Moury & Verdin, 2012). Historically, the genus *Tobamovirus* is particularly important as it consists of several devastating viral pathogens, including *Tobacco mosaic virus* (TMV), *Tomato mosaic virus* (ToMV), *Tomato mild mottle virus* (ToMMV), *Pepper mild mottle virus* (PMMoV), and *Cucumber green mottle mosaic virus* (CGMMV) (ICTV, 2021) In addition to these existing viral pathogens, newly emerging viral diseases also pose a serious threat to tomato and pepper production. Recently, *Tomato brown rugose fruit virus* (ToBRFV), a new virus in the genus *Tobamovirus*, has been identified from tomato plants (Luria et al., 2017; Salem et al., 2016). The virus has caused devastating disease outbreaks in tomato production areas in many countries, resulting in a severe reduction in yield (Avni et al., 2021; EPPO, 2020; Jones, 2021; Oladokun et al., 2019). Currently, ToBRFV is considered the most serious threat to tomato production in the world.

### 1.1 Discovery and distribution

ToBRFV was first isolated by Salem and colleagues in 2016 from greenhouse tomato plants grown in Jordan in April 2015 that showed typical viral symptoms (Salem et al., 2016). In this outbreak, the disease incidence was close to 100%. Although foliar symptoms were apparently mild at the end of the season, brown rugose symptoms on fruits were strong, which greatly affected fruit marketability. Subsequent diagnosis of these tomato plants with molecular biology and bioinformatics tools identified the causal agent to be a new tobamovirus and the virus was named *Tomato brown rugose fruit virus* (Salem et al., 2016). Shortly after this work, the Dombrovsky laboratory in Israel also reported the discovery of a new tobamovirus isolate from tomato plants grown in net houses in southern Israel in October–November of 2014 where the infected plant displayed mild to severe foliar symptoms and yellowing spots on fruits (Luria et al., 2017). A comprehensive molecular and morphological study was carried out to characterize the virus causing the outbreak. The Israeli isolate (GenBank accession no. KX619418) was found to share high sequence identity with the Jordanian isolate (KT383474). Thus, the first outbreak of ToBRFV was traced back to October 2014 in Israel. Moreover, Luria and co‐workers also discovered that ToBRFV could infect tomato cultivars carrying *Tm‐1*, *Tm‐2*, or *Tm‐2* ^*2*^ and infect pepper cultivars (Luria et al., 2017).

**FIGURE 1:**
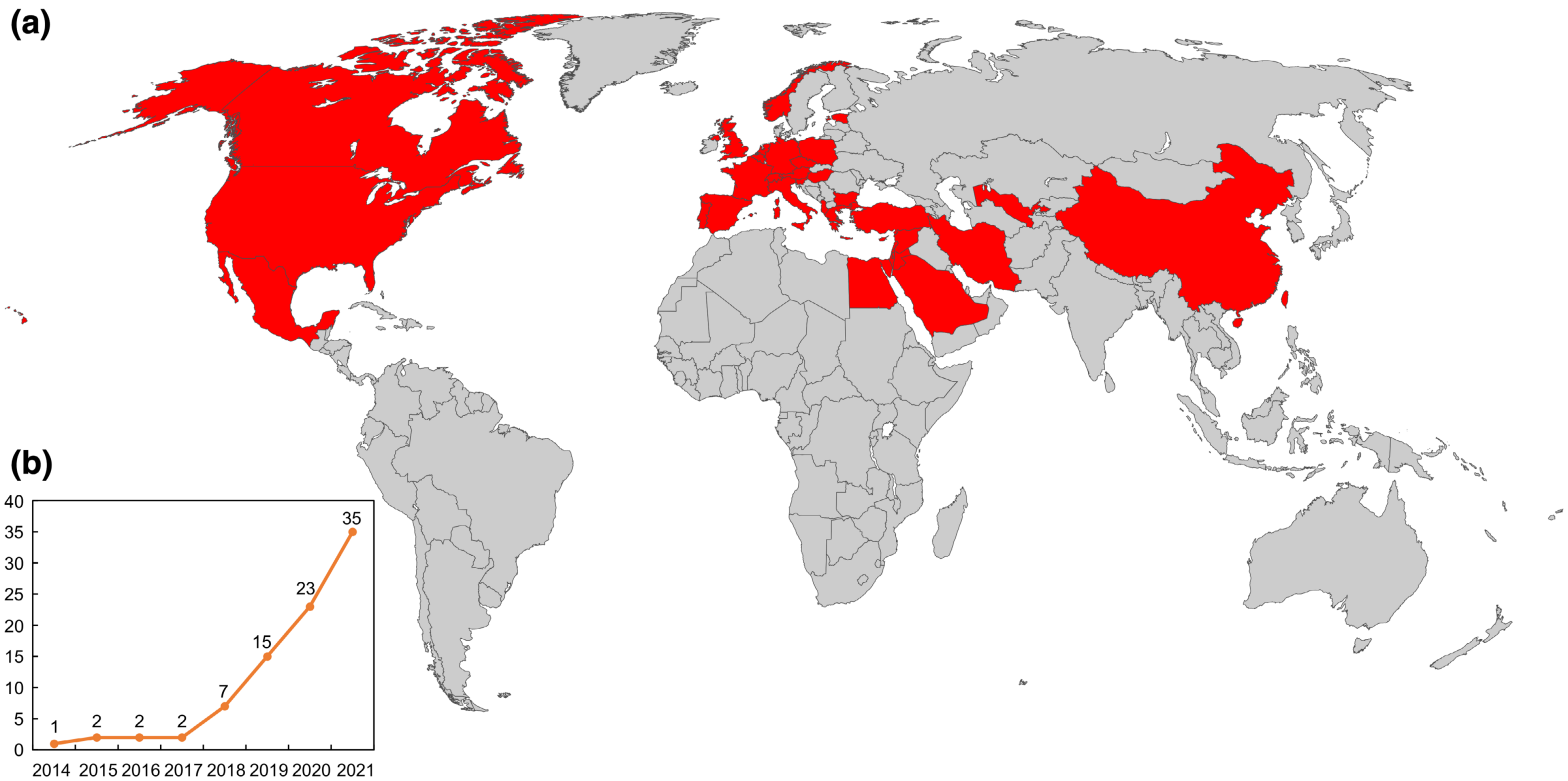
Distribution of ToBRFV. (a) The geographic map of ToBRFV. All countries with confirmed incidences are highlighted in red. (b) The graph shows the trend of the accumulated number of reported countries starting from the first outbreak in 2014. Data are adapted from the references in Table 1 and the European and Mediterranean plant protection organization (EPPO) global database (https://gd.eppo.int/taxon/TOBRFV/distribution).

After the discovery of ToBRFV in Jordan and Israel, the virus seemed to spread rapidly as the list of countries having ToBRFV has expanded very quickly. To date, the number has reached 35 across four continents, including Asia, Europe, North America, and Africa (Figure 1 and Table 1). As shown in Figure 1, these countries are mainly in the Middle East and Europe. Given the global nature of the seed production and distribution chain and ToBRFV's seed transmissibility, the extent of its spread is believed to be more severe than has been recorded. Consistent with this assumption, although the virus has not yet been officially reported from countries such as Australia, Peru, India, Ethiopia, and Japan, some tomato and pepper seeds exported from these countries to European and North American countries were found to be ToBRFV‐contaminated, suggesting that ToBRFV is highly likely to be present in these countries (EPPO, 2020, 2022).

**TABLE 1:** List of first reports of ToBRFV across the world

Continent	Country	Year of identification	Reference
Europe	Germany	2018	Menzel et al. (2019)
	Italy	2018	Panno et al. (2019a)
	Turkey UK Greece Netherlands Spain France Norway Albania Switzerland	2019 2019 2019 2019 2019 2020 2021 2022 2022	Fidan et al. (2019) Skelton et al. (2019) Beris et al. (2020) van de Vossenberg et al. (2020) Alfaro‐Fernández et al. (2021) Skelton et al. (2022) Hamborg and Blystad (2021) Orfanidou et al. (2022) Mahillon et al. (2022)
North America	USA	2018	Ling et al. (2019)
	Mexico	2018	Cambron‐Crisantos et al. (2018)
	Canada	2019	Sarkes et al. (2020)
Asia	Israel Jordan	2014 2015	Luria et al. (2017) Salem et al. (2016)
	Palestine	2018	Alkowni et al. (2019)
	China	2019	Yan et al. (2019)
	Syria	2020	Hasan et al. (2022)
	Lebanon	2020	Abou Kubaa et al. (2022)
	Iran	2021	Ghorbani et al. (2021)
	Saudi Arabia	2021	Sabra et al. (2021)
Africa	Egypt	2019	Amer and Mahmoud (2020)

Note

This list includes 23 countries with first reports published in peer‐reviewed journals. There are 12 additional countries with first reports to EPPO.

### 1.2 Transmission

In protected facilities such as greenhouses, ToBRFV is transmitted primarily by mechanical contact, including propagation materials, plant debris, contaminated soil, growing media, circulating water, workers' farming activities, and culture tools (Dombrovsky & Smith, 2017; Oladokun et al., 2019). Plants damaged by both abiotic and biotic factors may be more susceptible to tobamovirus infection (Dorokhov et al., 2018). Interestingly, the emission of methanol resulting from mechanical damage is likely to promote tobamovirus transmission between neighbouring plants (Dorokhov et al., 2012). Contaminated seeds are another major transmission mode because tobamoviruses are seedborne viruses (Dombrovsky & Smith, 2017). Tomato seeds extracted from ToBRFV‐infected fruits are 100% contaminated (Salem et al., 2022), although the virus is only detected externally on the seed coat (testa) (Klap et al., 2020a; Salem et al., 2022). Nevertheless, like all other seedborne viruses, the seed transmission rate from ToBRFV‐contaminated seeds to their seedling is low, ranging from 0.08% to 2.8% (Davino et al., 2020; Salem et al., 2022). These results suggest that seed transmission may establish some initial infection foci; further spread to cause an outbreak is through various types of mechanical contact.

International seed imports and exports are indispensable for global food security and sustainable agriculture (Dombrovsky & Smith, 2017). However, such international movement makes it possible for the long‐distance dissemination of seedborne viruses such as ToBRFV (Rizzo et al., 2021). For instance, several European countries, such as Spain and the Netherlands, have detected and intercepted some ToBRFV‐positive seed packages imported to their countries (EPPO, 2021). Furthermore, transportation of damaged ToBRFV‐contaminated fruits may also contribute to the long‐distance transmission of ToBRFV, albeit intact tomato fruits are not likely to transmit the virus (Davino et al., 2020; Klap et al., 2020a).

It is generally believed that there are no specific insect vectors that transmit ToBRFV (Oladokun et al., 2019). However, a recent study has shown that the bumblebee (*Bombus terrestris*), extensively used as a pollinator for tomato production, can transmit ToBRFV from hives carrying infectious inoculum to healthy tomato plants via buzz pollination (Levitzky et al., 2019). Thus, insect activities such as bumblebee pollination may accelerate the spread of the virus and bumblebees could be used to monitor greenhouses for the presence of ToBRFV. Panno and colleagues found that ToBRFV starting from two infected tomato plants could spread to an entire greenhouse, with an incidence rate of nearly 100%, in the presence of two bumblebee hives within a 9‐month monitoring period (Panno et al., 2020). As a proper control was lacking, the possible role of bumblebees in ToBRFV spread in this study needs to be confirmed. A more recent spatiotemporal investigation in tomato commercial greenhouses revealed that during a 24‐week culture period, ToBRFV was marginally aggregated at the initial stage, but vastly aggregated during the exponential phase of infection (González‐Concha et al., 2021). In this case, it was not clear if bumblebees were provided for pollination or not.

### 1.3 Host range

Based on published literature, ToBRFV can infect over 40 species belonging to four families: Amaranthaceae, Apocynaceae, Asteraceae, and Solanaceae (Table 2). Tomato and pepper plants are the only two crops that are the natural hosts of ToBRFV (Luria et al., 2017; Salem et al., 2016, 2020). In general, ToBRFV shares a very similar host range with ToMV and ToMMV (Chanda et al., 2021b). A striking difference is that ToBRFV can infect tomato cultivars harbouring *R* genes *Tm‐1*, *Tm‐2*, or *Tm‐2* ^*2*^ that confer resistance to tobamoviruses (Chanda et al., 2021b; EPPO, 2020; Luria et al., 2017). In pepper, *L* alleles mediate resistance to tobamoviruses such as TMV, ToMV, tobacco mild green mosaic virus (TMGMV), and PMMoV (Tomita et al., 2011). ToBRFV can infect pepper cultivars carrying *L* ^*1*^ or *L* ^*2*^ (Fidan et al., 2021). In pepper plants carrying *L* ^*3*^ and *L* ^*4*^, inoculation with ToBRFV triggers a hypersensitive response (HR), a typical resistance response (Fidan et al., 2021). Like other tobamoviruses, ToBRFV infects *Nicotiana benthamiana*, *N. glutinosa*, and *N. tabacum* in the family Solanaceae and *Chenopodium quinoa* in the family Amaranthaceae but does not infect *Arabidopsis thaliana* or *Brassica rapa* in the family Brassicaceae as well as representative species from Cucurbitaceae and Verbenaceae families (Chanda et al., 2021b; Yan et al., 2021b). Experimental hosts are important tools for plant molecular biologists. *N. benthamiana* has been recognized as an ideal model plant to study plant–microbe interactions (Bally et al., 2018). Indeed, the model plant *N. benthamiana* has already been used to study ToBRFV in several recent publications (Hak & Spiegelman, 2021; Ma et al., 2021; Yan et al., 2021a).

**TABLE 2:** Host plants reported of tomato brown rugose fruit virus

Scientific name	Symptoms^a^		Virus detection^b^	References
	Local reaction	Systemic reaction		
*Solanum lycopersicum*	NS	DGB, LC, LD, LM, LN, LR, LY, M, SS	+	Luria et al. (2017), Menzel et al. (2019) Salem et al. (2016), Yan et al. (2021a)
*Capsicum annuum*	LY, N, NL, NRS	C, LM, M, NRS, PS, SN	+	Luria et al. (2017) Salem et al. (2020) Yan et al. (2021a)
*Catharanthus roseus*	NS	NS	+	Chanda et al. (2021b)
*Chenopodium album*	CLL, NRS	C, LD	+	Chanda et al. (2021b)
*Chenopodium amaranticolor*	NL	NS	+	Luria et al. (2017)
*Chenopodium berlandieri*	CLL, NRS	NS	−	Chanda et al. (2021b)
*Chenopodium giganteum*	NRS	NS	−	Chanda et al. (2021b)
*Chenopodium murale*	CLL, NLL	M	+	Luria et al. (2017), Salem et al. (2016)
*Chenopodium quinoa*	CLL, NL, NRS	LD, LM	+	Chanda et al. (2021b), Luria et al. (2017), Salem et al. (2016)
*Datura metel*	NLL	NS	+	Salem et al. (2016)
*Datura stramonium*	NLL	NS, PD	+	Chanda et al. (2021b), Luria et al. (2017), Salem et al. (2016)
*Emilia sonchifolia*	NLL	VN	+	Chanda et al. (2021b), Luria et al. (2017)
*Glebionis coronaria*	NS	LM, M	+	Chanda et al. (2021b)
*Gomphrena globosa*	NS	LM	+	Chanda et al. (2021b)
*Nicotiana benthamiana*	N, NL	LC, LM, LY, M, PC, PD, PS	+	Chanda et al. (2021b), Luria et al. (2017), Salem et al. (2016), Yan et al. (2021a)
*Nicotiana clevelandii*	NL	LY, NL	+	Luria et al. (2017)
*Nicotiana debneyi*	NS	LM	+	Chanda et al. (2021b)
*Nicotiana glutinosa*	N, NLL	LM, M, PD	+	Chanda et al. (2021b), Luria et al. (2017), Salem et al. (2016)
*Nicotiana megalosiphon*	NLL	LM, M	+	Salem et al. (2016)
*Nicotiana occidentalis*	LM	M	+	Luria et al. (2017)
*N. occidentalis* subsp*. hesperis*	NRS	CP, LM, N	+	Chanda et al. (2021b)
*Nicotiana rustica*	CLL, NRS	LD, LM, PS	+	Chanda et al. (2021b)
*Nicotiana sylvestris*	NL	M	+	Luria et al. (2017)
*Nicotiana tabacum* ‘Samsun’	CLL, M	CP, LM, N	+	Chanda et al. (2021b), Luria et al. (2017), Salem et al. (2016)
*N. tabacum* ‘Samsun NN’	HR, NL	NS	−	Luria et al. (2017), Yan et al. (2021a)
*N. tabacum* ‘White Burley’	CLL	LM	+	Salem et al. (2016)
*N. tabacum* ‘Xanthi nc’	NLL	PD	+	Chanda et al. (2021b)
*N. tabacum* ‘Zhongyan 102’	NS	NS	+	Yan et al. (2021a)
*Petunia* × *hybrida*	NS	LM	+	Chanda et al. (2021b), Luria et al. (2017)
*Physalis angulata*	N	NL	+	Chanda et al. (2021b)
*Physalis pubescens*	N	NL	+	Chanda et al. (2021b)
*Solanum arcanum*	NA	B, LC, LD, LM, LR, LT, SS	+	Jewehan et al. (2022)
*Solanum cheesmaniae*	NA	B, LC, LD, LM, LR, SS	+	Jewehan et al. (2022)
*Solanum chilense*	NA	B, LC, LD, LM, LR, SS	+	Jewehan et al. (2022)
*Solanum chmielewskii*	NA	LC, LM, LR, SS	+	Jewehan et al. (2022)
*Solanum corneliomulleri*	NA	B, LC, LD, LM, LR, SS	+	Jewehan et al. (2022)
*Solanum galapagense*	NA	B, LC, LD, LM, LR, SS	+	Jewehan et al. (2022)
*Solanum habrochaites*	NA	B, LC, LD, LM, LR	+	Jewehan et al. (2022)
*Solanum huaylasense*	NA	LD, LM, LT	+	Jewehan et al. (2022)
*Solanum juglandifolium*	NA	LM	+	Jewehan et al. (2022)
*Solanum melongena*	NS	NS	+	Yan et al. (2021a)
*Solanum neorickii*	NA	B, LD, LM, LR, SS	+	Jewehan et al. (2022)
*Solanum nigrum*	CLL, NRS	LM, M	+	Chanda et al. (2021b), Luria et al. (2017)
*Solanum ochranthum* ^c^	NA	LM	+	Jewehan et al. (2022)
*Solanum pennellii*	NA	LC, LD, LM	+	Jewehan et al. (2022)
*Solanum peruvianum*	NA	B, LC, LD, LM, LR, SS	+	Jewehan et al. (2022)
*Solanum pimpinellifolium*	NA	B, LC, LD, LM, LR, SS	+	Jewehan et al. (2022)
*Solanum sitiens*	NA	LM	+	Jewehan et al. (2022)
*Solanum tuberosum* ‘Kexin 1’	NS	NS	+	Yan et al. (2021a)

^a^ Symptoms observed on local and systemic leaves: B, bubbles; C, chlorosis; CLL, chlorotic local lesion; CP, chlorotic plant; DGB, dark green bulges; HR, hypersensitive reaction; LC, leaf curling; LD, leaf deformation; LM, leaf mosaic; LN, leaf narrowing; LR, leaf rolling; LT, leaf twisted; LY, leaf yellowing; M, mottling; N, necrosis; NA, not applicable; NL, necrotic lesions; NLL, necrotic local lesions; NRS, necrotic ringspot; NS, no symptoms; PC, plant collapse; PD, plant death; PS, plant stuntedness; SN, stem necrosis; SS, shoestring; VN, vein necrosis.

^b^ Reverse transcription‐PCR or ELISA detection on upper systemic leaves: +, positive; −, negative.

^c^ Three *S. ochranthum* accessions (LA2160, LA2162, and LA2166) are highly resistant to ToBRFV infection (Jewehan et al., 2022).

### 1.4 Disease symptoms

**FIGURE 2:**
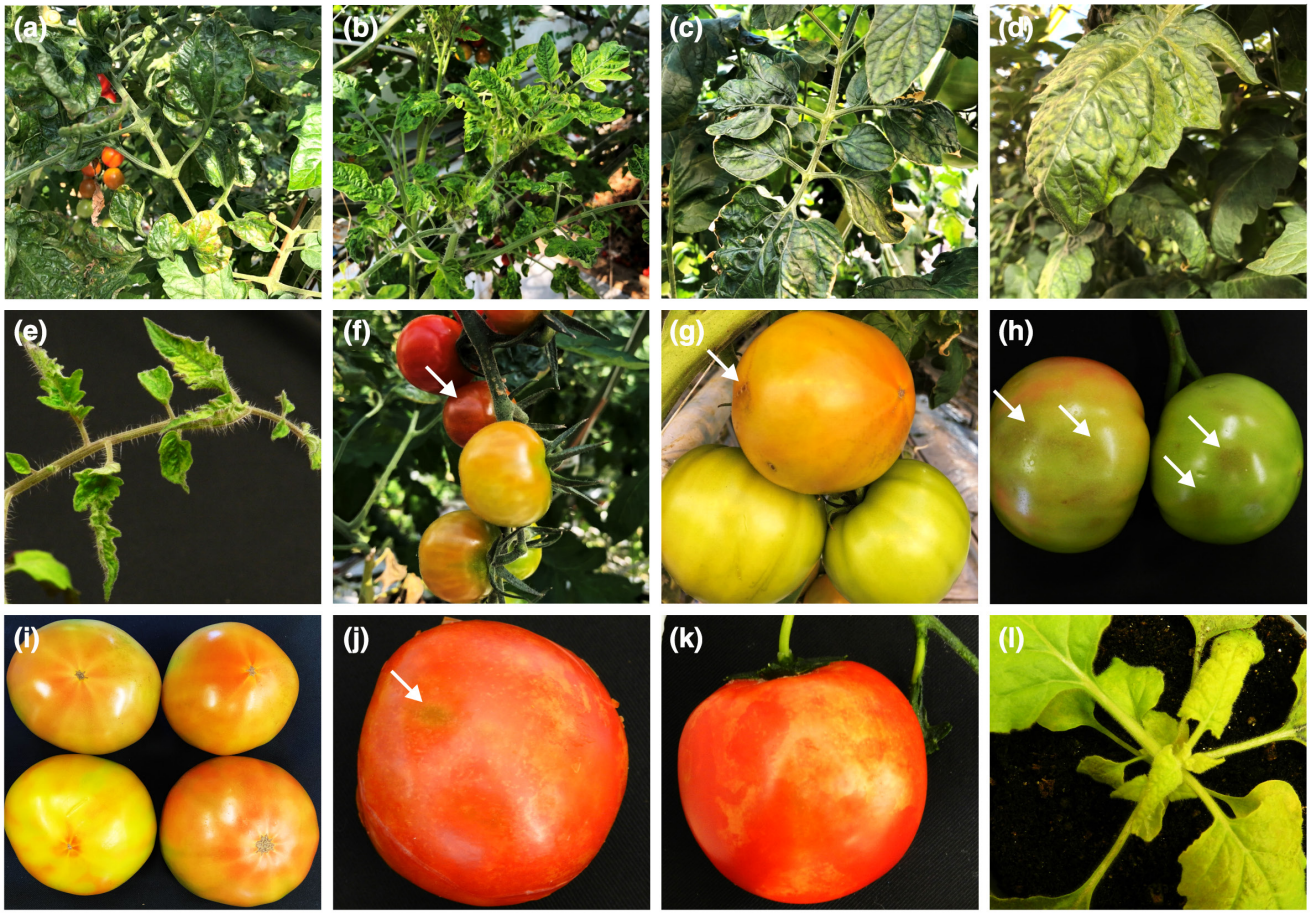
Typical symptoms induced by ToBRFV. (a–e) Severe mosaic, chlorotic mottling, necrotic, deformed, dark green bulges, and narrowing symptoms on leaves of tomato cultivars Piccolo (a, b), Kivu (c, d), and Moneymaker (e). (f–k) Fruits with brown rugosity (white arrows), yellowing, yellow patches, marbling, and deformation symptoms on tomato cultivars Piccolo (f, g), Kivu (h, i), and Moneymaker (k). (l) ToBRFV‐infected *Nicotiana benthamiana* showing yellowing and curling leaves along with the stunting phenotype.

Symptoms resulting from ToBRFV infection are very similar to those from other tobamoviruses such as ToMV (Alon et al., 2021). Virus‐induced foliar symptoms are more obvious in young leaves at the top of plants. Typical symptoms on tomato include mosaic, chlorotic, mottling, and deformed leaves, and necrotic spotted or brown rugose fruits. Symptom severity may vary among different cultivars, plants, growth stages, and culture conditions (Figure 2). For instance, fruits of greenhouse tomato plants infected with the Jordanian isolate displayed strong brown rugosity on fruit, while in contrast foliar symptoms were found to be mild (Salem et al., 2016). Infected tomato plants grown in net houses in southern Israel showed mild to severe mosaic symptoms on leaves and 10%–15% of fruits from diseased plants were yellow‐spotted (Luria et al., 2017). Foliar symptoms such as leaf narrowing, chlorotic mottling, and dark green bulges were documented in reports from Germany and the Netherlands (Menzel et al., 2019; van de Vossenberg et al., 2020), and drying and brown necrosis patches on the pedicles, calyces, and flowers were observed in the United States and China (Chanda et al., 2021b; Yan et al., 2019). Infection by ToBRV can reduce fruit yield by 15%–55% regardless of whether or not tested tomato cultivars carry the resistance gene *Tm‐2* ^*2*^ (Avni et al., 2021).

In pepper plants, ToBRFV infection often induces mosaic, mottling, yellowing, and necrotic lesions on leaves, very similar to those seen on tomato plants, while fruits exhibit small yellow to brown rugose dots and necrotic blotches (Alkowni et al., 2019; Chanda et al., 2021b; Fidan et al., 2021; Salem et al., 2020). The infected young pepper plants become dwarfed and stunted, sometimes with necrotic or brown stems (Abou Kubaa et al., 2022; Yan et al., 2021b). In *N. benthamiana*, ToBRFV infection causes distinct narrowing, yellowing, and curling in upper noninoculated leaves, eventually leading to plant collapse and die‐off (Ma et al., 2021; Yan et al., 2021b).

### 1.5 Mixed infection

Mixed infections by multiple plant viruses are common in nature and these viruses may interact with each other in a synergistic or an antagonistic fashion (Syller, 2012). So far, only synergistic interactions have been observed in ToBRFV‐involved mixed infections. *Tomato spotted wilt virus* (TSWV) is another important tomato‐infecting virus and the disease caused by this virus is one of the most destructive diseases in tomato production (Scholthof et al., 2011). Mixed infections of ToBRFV and TSWV result in more severe symptoms on tomato fruits (Luria et al., 2017). Mixed infections are also common on pepper (Luria et al., 2018). For instance, new symptoms occurred in pepper coinfected by ToBRFV and paprika mild mottle virus, another tobamovirus. The potexvirus *Pepino mosaic virus* (PepMV) is also a major tomato virus that impacts tomato production worldwide (Hanssen et al., 2010). One of the major PepMV control strategies is cross‐protection by preinoculation of attenuated mild PepMV strains, which is widely used in greenhouse tomato production. However, a recent study showed that tomato plants coinfected by ToBRFV and a mild PepMV strain CH2 induced severe new viral disease symptoms including open or scarred unripe fruits and various leaf phenotypes such as bubbling, yellow patches, narrowing or serrated margins (Klap et al., 2020b). By sequential inoculations of tomato plants with ToBRFV and the PepMV mild isolate, they found that preinoculation of ToBRFV enhanced PepMV titres and induced symptoms characteristic of PepMV aggressive strains (Klap et al., 2020b). Moreover, when fruits infected by ToBRFV and PepMV were damaged, they could serve as an effective inoculum source (Klap et al., 2020a). These observations raise serious concerns about the application of mild PepMV strains to cross‐protect against severe PepMV when ToBRFV is endemic.

## 2 GENOME ORGANIZATION AND SEQUENCE DIVERSITY

**FIGURE 3:**
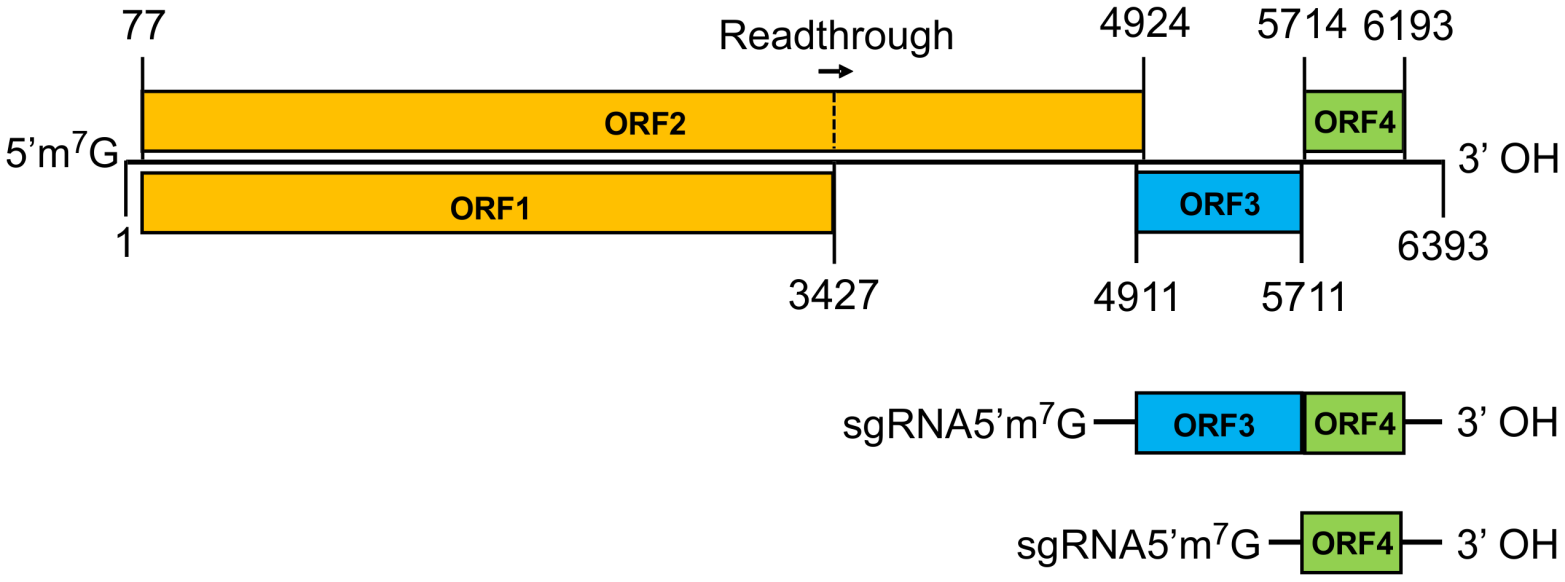
Schematic representation of the ToBRFV genome. The numbers indicate the nucleotide positions where each open reading frame (ORF) begins and ends in the representative ToBRFV isolate (NC_028478.1, 6393 bp). ORF2 is translated via readthrough of ORF1 at nucleotide 3427. The lower panel shows that ORF3 and ORF4 are expressed from subgenomic RNAs.

The ToBRFV genome consists of a single‐stranded, positive‐sense RNA of approximately 6.4 kb in length that is encapsulated into crinkled cylindrical virions c.300 nm long and 18 nm in diameter (Dombrovsky & Smith, 2017; Luria et al., 2017; Oladokun et al., 2019). As mentioned above, ToBRFV is a member of the genus *Tobamovirus*. *Tobamovirus* is composed of 37 formal species, representing the largest genus among seven genera in the family *Virgaviridae* (ICTV, 2021). TMV, the first virus of all the kinds discovered, is the type member of the genus (Knapp & Lewandowski, 2001). A typical tobamoviral genome encompasses four open reading frames (ORF1 through ORF4), with a 7‐methylguanosine 5′ triphosphate cap at the 5′ terminus and three consecutive pseudoknots followed by a transfer RNA‐like structure at the 3′ untranslated region (Dorokhov et al., 2018; ICTV, 2021; Ishibashi & Ishikawa, 2016; Knapp & Lewandowski, 2001). ORF1 and ORF2 are translated directly using the genomic RNA, whereas ORF3 and ORF4 are expressed from subgenomic RNAs (Figure 3; ICTV, 2021; Ishibashi & Ishikawa, 2016; Oladokun et al., 2019). More specifically, the 126‐ and 183‐kDa proteins encoded by tobamoviral ORF1 and ORF2 (expressed via readthrough at an amber stop codon UAG downstream of OFR1), respectively, are replicase proteins and participate in virus genome replication. The 126‐kDa protein can also act as an RNA silencing suppressor. The ORF3‐encoded 30‐kDa is a movement protein (MP) that is essential for viral cell‐to‐cell movement. Two recent studies have shown that ToBRFV MP is the key factor for overcoming *Tm‐2* ^*2*^‐mediated resistance (Hak & Spiegelman, 2021; Yan et al., 2021a). The coat protein (CP) encoded by ORF4 has a predicted mass of 17.5 kDa and is involved in viral particle assembly and long‐distance movement (Ishibashi & Ishikawa, 2016).

**FIGURE 4:**
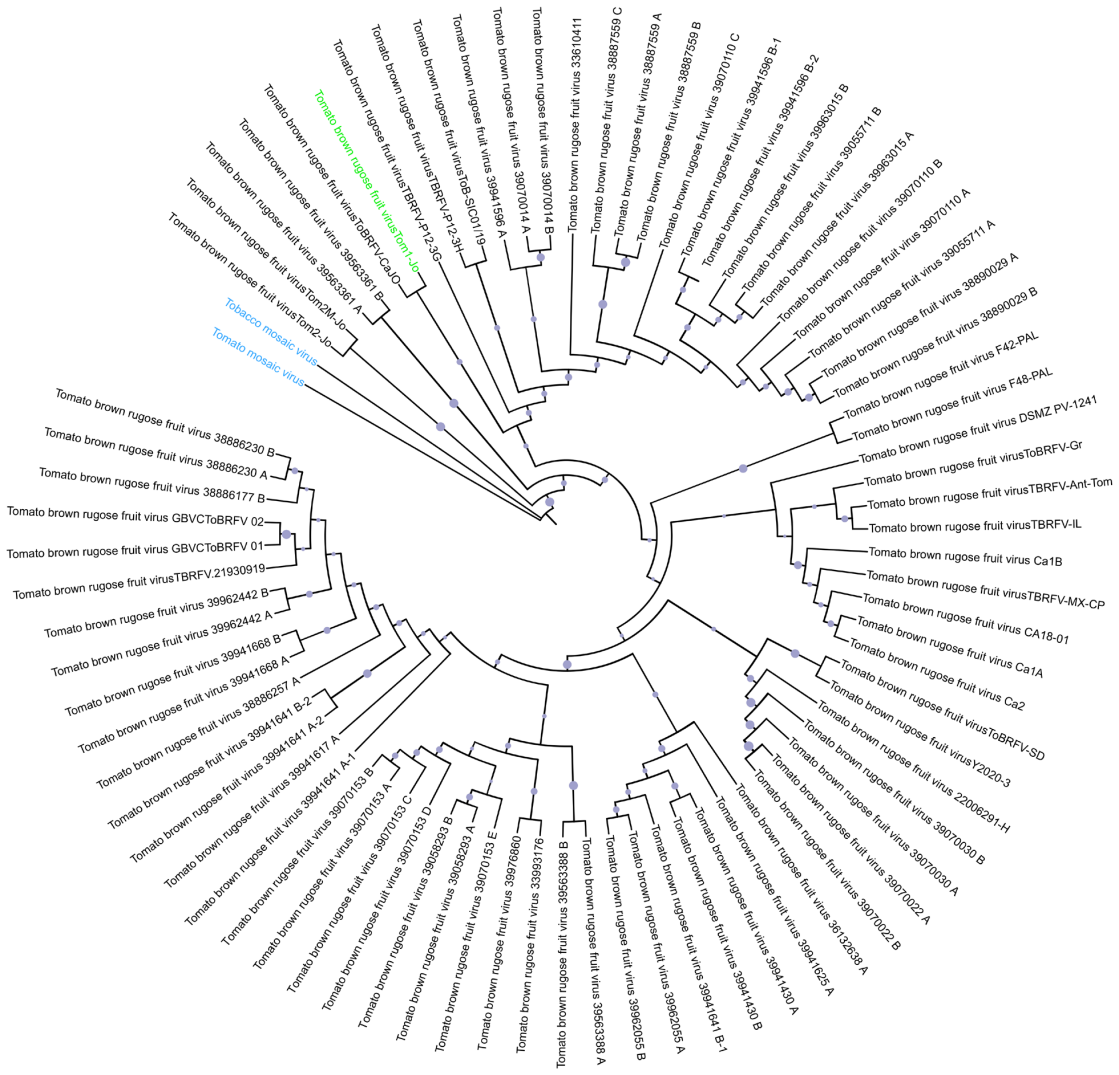
Neighbour‐joining phylogenetic tree based upon the complete genome sequences of ToBRFV, TMV, and ToMV. TMV (NC_001367.1), and ToMV (NC_002692.1) are used as outgroups (shown in blue). The representative ToBRFV isolate (NC_028478.1) is highlighted in green. The bootstrap confidence values generated by 1000 replications are shown in purple at the branches.

As of March 2022, 78 full‐length ToBRFV genome sequences have been deposited in the National Center for Biotechnology Information (NCBI) database. We retrieved these sequences and conducted a phylogenetic analysis to explore their possible relationship and diversity. A phylogenetic tree was constructed based on nucleotide sequences (Figure 4). All ToBRFV isolates are clearly distinct to TMV and ToMV. ToBRFV isolates are grouped into three main clusters, which is in line with previous results (van de Vossenberg et al., 2020). Isolates from three European countries, including various isolates from the Netherlands (such as 38886230, 39962442, and 39941668), Belgium (GBVC ToBRFV 01 and 02) and the UK (TBRFV.21930919) form one cluster, whereas five North American isolates (CA18‐01, Ca1A, Ca1B, Ca2, and TBRFV‐MX‐CP) from the USA, Canada, and Mexico form another cluster. The finding that isolates from the same global geographic region exhibit a high degree of similarity and are grouped into the same cluster raises the possibility that they share the same origin, for example from the same source/lot of contaminated seeds.

## 3 DETECTION AND DIAGNOSIS

Because ToBRFV is highly transmissible and poses an emerging threat to the global tomato industry, rapid and accurate detection and diagnosis are particularly critical for the implementation of timely measures to control its spread. PCR (for detection of DNA viruses) or reverse transcription PCR (RT‐PCR, for detection of RNA viruses) and enzyme‐linked immunosorbent assay (ELISA) are the most commonly used methods to detect viruses. Transmission electron microscopy (TEM) and next‐generation sequencing (NGS) followed by data analysis have also been used to investigate the presence of ToBRFV. Due to similar virion morphology and serological cross‐reactivity (with commercially available antibodies), the TEM and commercial ELISA kits currently available cannot distinguish ToBRFV from other tobamoviruses. NGS approaches have been used successfully to detect ToBRFV and have been instrumental in determining full genomic sequences of ToBRFV, and useful for understanding evolutionary relationships and the spread of ToBRFV worldwide. However, NGS methods are not the preferred choice for ToBRFV detection due to the costs (monetary, expertise, and infrastructure) and processing time required compared to amplification‐based approaches.

Several nucleic acid amplification‐based protocols, including RT‐quantitative PCR (RT‐qPCR) and droplet digital PCR (ddPCR), have been developed to detect ToBRFV in leaf and seed samples (Chanda et al., 2021b; Fidan et al., 2021; Luigi et al., 2022; Menzel & Winter, 2021; Panno et al., 2019b; Vargas‐Hernández et al., 2022). Yan et al. (2021b) reported a quadruplex RT‐PCR protocol that allows for the simultaneous detection of four tomato‐infecting viruses, ToBRFV, TMV, ToMV, and TSWV, from the same sample in one reaction. One more amplification‐based assay detection method to effectively detect ToBRFV in infected plants or contaminated seeds is loop‐mediated isothermal amplification (LAMP) PCR (Rizzo et al., 2021; Sarkes et al., 2020). Because this assay can be used to directly visualize positive results without the requirement of specialized equipment, infrastructure, and/or expertise, it can be used for laboratory tests or in a field setting. The LAMP PCR may be further combined with clustered regularly interspaced short palindromic repeat (CRISPR)/CRISPR‐associated protein (Cas) technology to introduce CRISPR RNAs (crRNAs) from tobamoviruses such as ToMV and ToBRFV for species‐specific detection with in‐field applicability (Alon et al., 2021; Bernabé‐Orts et al., 2022).

As ELISA is a fast, sensitive, and cost‐effective approach for virus detection and diagnosis, efforts have been made to solve the specificity issue due to cross‐reaction with other tobamoviruses. Two monoclonal antibodies that sensitively and specifically recognize ToBRFV CP without serological cross‐reactions with TMV and ToMV have been developed (Bernabé‐Orts et al., 2021). Moreover, a colloidal gold immunochromatographic strip prepared with double monoclonal antibodies against the CP was shown to be capable of producing precise results in 5 min without any cross‐reactions (Yan et al., 2022). The availability of these ToBRFV‐specific antibodies paves the way for the development and commercialization of ToBRFV‐specific ELISA kits or test strips in the near future.

Several nucleic acid amplification‐based detection techniques have been included in the standard diagnostic procedure for ToBRFV by different organizations. For example, the standard method for detection of ToBRFV in tomato and pepper seed developed by ISF‐ISHI‐Veg (International Seed Federation/International Seed Health Initiative for Vegetables) contains a detailed protocol for seed sampling, RNA extraction, and a one‐step duplex RT‐qPCR (the seed extract qPCR assay, SE‐qPCR) (ISF, 2020). As SE‐qPCR detects both infectious and noninfectious ToBRFV genetic material, the ISF‐ISHI‐Veg procedure also includes a bioassay to confirm the presence or absence of infectious ToBRFV. The diagnostic procedure for ToBRFV in plant material and in seeds recommended by the European and Mediterranean Plant Protection Organization (EPPO) contains sample preparation and RNA extraction protocols from plant leaves, fruits or seeds, and four PCR‐based molecular tests (EPPO, 2021), including two one‐step conventional RT‐PCR protocols (Alkowni et al., 2019; Roodríguez‐Mendoza et al., 2019), SE‐qPCR of the ISF‐ISHI‐Veg procedure, and an RT‐qPCR protocol (Menzel & Winter, 2021). The choice of molecular tests will depend on the expected virus titre in the samples to be tested. All molecular tests may be used for detection of ToBRFV in leaf samples, which usually contain high levels of the virus. For ToBRFV‐contaminated seeds in which lower viral titres are expected, SE‐qPCR and RT‐qPCR tests are preferred. It should be noted that the recommended diagnostic procedures may be updated along with the ongoing development and validation of novel detection techniques/tools.

## 4 MP IS RESPONSIBLE FOR OVERCOMING *TM‐2* ^*2*^‐MEDIATED RESISTANCE

Several *R* genes, including *N*, *Tm‐1*, *Tm‐2*, *Tm‐2* ^*2*^, *L* ^*1*^, *L* ^*2*^, *L* ^*3*^, and *L* ^*4*^, have been identified and deployed into solanaceous crop species (tomato, pepper, and tobacco) through breeding programmes to control tobamoviruses (de Ronde et al., 2014). *Tm‐1* encodes a triosephosphate isomerase (TIM) barrel‐like protein, and all the other seven *R* genes encode nucleotide‐binding domain leucine‐rich repeat proteins (NB‐LRRs). *Tm‐2*, *Tm‐2* ^*2*^, and the four *L* genes encode coiled‐coil NB‐LRR (CC‐NB‐LRR), whereas *N* codes for Toll interleukin‐1 receptor‐NB‐LRR (TIR‐NB‐LRR). On tobamovirus infection, these *R*‐encoded proteins recognize specific proteins of the target virus, termed avirulence effectors (Avr), to activate effector‐triggered immunity response (ETI) to restrict virus infection. The tobamoviral CP is the Avr effector of *L*‐mediated resistance to tobamoviruses and the LRR domain of L proteins is the determinant of recognition spectra (Tomita et al., 2011). The *N*‐encoded TIR‐NB‐LRR indirectly recognizes its Avr, TMV P50 (the 50 kDa helicase domain within the 126‐kDa replicase) through its TIR domain that directly interacts with the N receptor‐interacting protein NRIP1 and the P50 domain to trigger HR and restrict TMV infection (Caplan et al., 2008; Zhang et al., 2019). The CC‐NBS‐LRR encoded by *Tm‐2* ^*2*^ perceives tobamovirus infection by recognizing the tobamoviral 30 kDa MP to activate immune signalling through its C‐terminal LRR domain (Kobayashi et al., 2011; Weber & Pfitzner, 1998). The *Tm‐2* ^*2*^ gene, originally identified from the wild tomato species *S. peruvianum*, is a well‐known durable dominant *R* gene that has remained effective against a variety of tobamoviruses, including the notorious TMV and ToMV, for over five decades. In most cases, *Tm‐2* ^*2*^ confers extreme resistance (ER) without visible HR (necrotic lesion) (Chanda et al., 2021b; Lanfermeijer et al., 2003; Zhang et al., 2013). Transgenic *N. benthamiana* plants carrying the *Tm‐2* ^*2*^ gene also show ER to tobamoviruses (Zhang et al., 2013).

To explore the underlying mechanism by which ToBRFV breaks down *Tm‐2* ^*2*^‐mediated resistance, Maayan et al. (2018) conducted a comparative genomic analysis and identified 12 potential resistance‐breaking mutations in the 30 kDa MP and nine in the 126‐kDa replicase. Hak and Spiegelman (2021) performed transient expression assay and found that ToBRFV MP fails to trigger *Tm‐2* ^*2*^‐mediated HR. Through a gain‐of‐function experiment, these authors showed that a chimeric ToMV infectious clone, in which the MP was replaced by the ToBRFV MP, was able to infect tomato plants harbouring *Tm‐2* ^*2*^. Using a hybrid protein approach, they further determined that the amino acids 1 to 266 rather than the C‐terminal region of the ToBRFV MP is responsible for breakdown of *Tm‐2* ^*2*^ resistance (Hak & Spiegelman, 2021). Concomitantly and independently, Yan et al. (2021a) conducted both loss‐of‐function and gain‐of‐function assays using chimeric TMV and ToBRFV infectious clones where the MP coding gene was reciprocally swapped, and concluded that the ToBRFV MP is the virulence determinant for ToBRFV to infect *Tm‐2* ^*2*^ plants. They further mapped the region of ToBRFV MP necessary for breakdown of *Tm‐2* ^*2*^ resistance to the central domain (amino acids 60–186) (Yan et al., 2021a). Through the introduction of point mutations into this domain, they identified six residues, H67, N125, K129, A134, I147, and I168, to be critical for overcoming *Tm‐2* ^*2*^‐mediated resistance (Yan et al., 2021a). These results advance our understanding of how ToBRFV overcomes the durable resistance mediated by *Tm‐2* ^*2*^. However, it still remains unknown if and how the central region and the six residues affect the recognition of ToBRFV MP by the *Tm‐2* ^*2*^‐encoded CC‐NBS‐LRR and the downstream activities in the signalling cascade of this ETI.

As mentioned above, ToBRFV can infect pepper cultivars that carry *L* ^*1*^ or *L* ^*2*^ genes systemically, whereas pepper plants harbouring *L* ^*3*^ or *L* ^*4*^ are resistant to ToBRFV as infection with ToBRFV triggers HR (necrotic lesions) (Fidan et al., 2021). These data suggest that ToBRFV can overcome *L* ^*1*^‐ and *L* ^*2*^‐mediated resistance. Fidan et al. (2021) also demonstrated that under higher temperatures, infection by the Turkey isolate becomes systemic on *L* ^*3,4*^ pepper plants. Therefore, resistance genes *L* ^*3*^ and *L* ^*4*^ may operate conditionally against tobamoviruses. More studies are needed to better understand differential responses in *L* ^*1,2*^ and *L* ^*3,4*^ pepper plants to ToBRFV and the molecular mechanism underlying these responses.

## 5 CONTROL STRATEGIES

### 5.1 Quarantine and phytosanitary measures

Currently, there are no chemicals that can be used to cure ToBRFV‐infected plants and there are no resistant cultivars commercially available. As discussed above, ToBRFV infection severely reduces fruit yields and quality. When outbreaks occur, growers are often forced to terminate the crop. In view of such catastrophic losses caused by ToBRFV, many regions, countries, and international organizations have implemented regulation measures. In November 2018, the California Department of Food and Agriculture (CDFA) evaluated the risk of ToBRFV and proposed a rating A for this virus (CDFA, 2018). In November 2019, the United States Department of Agriculture (USDA) issued a Federal Order imposing restrictions on imports of tomato and pepper seed lots, transplants, and fruit from all countries where ToBRFV exists (USDA Federal Order, 2019). In June 2020, USDA amended the order for the import requirements for tomato and pepper fruit for consumption (USDA Federal Order, 2020). To prevent the introduction into and spread within the European Union (EU), the EU announced the Commission Implementing Decision 2019/1615 on emergency measures (EU, 2019), and then repealed this decision and declared the Commission Implementing Regulation 2020/1191 in August 2020 (EU, 2020). EPPO added ToBRFV into the EPPO Alert List in January 2018, and then updated to the EPPO A2 list of pests and recommended it for regulation as a quarantine pest in 2020 (EPPO, 2020, 2021). In July 2021, China also declared a quarantine status on ToBRFV and notified World Trade Organization members of the phytosanitary requirements of tomato and pepper seeds exported to China (China, 2021). Such timely regulation measures are expected to prevent or least slow down the introduction into and spread in the countries/regions where ToBRFV has not been detected or widely spread yet.

As ToBRFV is a seedborne virus and the introduction of this virus into uninfected areas is believed to occur through contaminated seeds, it is highly recommended to use virus‐free tomato and pepper seeds that are harvested from healthy parental plants (Salem et al., 2022). Disinfectant treatment of seeds has been suggested as a preventative measure against ToBRFV. Based on several recent studies (Chanda et al., 2021a; Davino et al., 2020; Samarah et al., 2021), effective disinfection chemicals include 0.5% or 2% Virocid, 0.5% lactoferrin, 10% Clorox, 3% Virex, 2% hydrochloric acid, 10% trisodium phosphate, 4% hydrogen peroxide, and 2.5% sodium hypochlorite. Heat treatment at 72°C for 72 h seems ineffective (Samarah et al., 2021).

In addition to using healthy seeds, the production site should be ToBRFV‐free. As ToBRFV is very stable and highly contagious, crop rotation helps but has limited effect. If infection occurs, eradication of infected and suspicious plants should be done as quickly and thoroughly as possible. Planting instruments such as knives, containers, trays, carts, and irrigation pipes may be disinfected with sodium hypochlorite solution (Smith & Dombrovsky, 2019). Greenhouse workers and staff should follow proper hygiene protocols. Bumblebee hives may be reservoirs of ToBRFV primary inoculum, and timely detection of ToBRFV and replacement, if required, of hives are suggested (Levitzky et al., 2019).

### 5.2 Cross‐protection with attenuated variants

Cross‐protection is a promising, potent proactive approach for the control of ToBRFV. Essentially, the concept was developed based on the discovery a century ago that preinfection with an attenuated variant of TMV protects tobacco plants against a severe TMV strain (Pechinger et al., 2019; Wagemans et al., 2022; Ziebell & Car, 2010; Ziebell & MacDiarmid, 2017). The possible molecular mechanisms, that is, RNA silencing and exclusion, have been extensively investigated. Cross‐protection has been demonstrated to be an effective, practical approach for the control of many plant viruses, including two tobamoviruses (TMV and CGMMV) (Ali et al., 2016; Goto et al., 1984, 1997), *Citrus tristeza virus* (Costa & Muller, 1980), *Cucumber mosaic virus* (Ziebell et al., 2007), *Papaya ringspot virus* (Yeh & Gonsalves, 1984), PepMV (Chewachong et al., 2015; Hanssen & Thomma, 2010), and *Zucchini yellow mosaic virus* (Cho et al., 1992). A representative example is CGMMV. Widespread CGMMV epidemics, enhanced with the difficulty of disinfecting contaminated greenhouses and the additional introduction of the new virus each year via contaminated seed, is a major problem for indoor cucurbit crop production worldwide. An attenuated strain (SH33b) of CGMMV, derived from its parental severe strain SH through ultraviolet irradiation, has been used for the effective control of CGMMV in greenhouse muskmelon in Japan (Motoyoshi & Nishiguchi, 1988). With the availability of powerful molecular biology and bioinformatics tools, the development of attenuated ToBRFV variants in a relatively short time frame is possible. Essentially, one can construct an infectious clone, conduct comparative genome analysis of the ToBRFV genome sequence with other attenuated tobamoviruses to find potential nucleotides of interest, introduce substitution mutations into these, and finally examine their suitability for cross‐protection.

### 5.3 Genetic resistance

Genetic resistance represents the most effective, economical, and sustainable approach in the control of viral diseases, as it is environmentally friendly, target‐specific, and provides reliable protection without additional labour or material costs during the growing season (Nicaise, 2014; Wang, 2015). Unfortunately, no ToBRFV‐resistant cultivars are currently commercially available. Tomato germplasms, particularly its close relatives, are important sources of genetic resistance to viruses. Previously, *Tm‐1*, *Tm‐2*, and the durable tobamovirus‐resistant gene *Tm‐2* ^*2*^ were all identified from wild tomatoes (*Tm‐1* from *S. habrochaites*, *Tm‐2* and *Tm‐2* ^*2*^ from *S. peruvianum*) (Hall, 1980; Lanfermeijer et al., 2003; Pelham, 1966). These genes were introgressed into cultivated tomatoes by breeders over several generations. Screening of 636 *Solanum* accessions from sections *Lycopersicon* and *Juglandifolia* resulted in the identification of three *S. ochrantum* accessions (LA2160, LA2688, and LA1385) highly resistant to ToBRFV (Jewehan et al., 2022). As these three accessions can also restrict TMV and ToMV to inoculation foci, they have great potential as a source of genetic resistance to tobamoviruses for tomato breeding programmes. Another screening of 160 genotypes by Zinger and colleagues identified one resistant and 29 tolerant genotypes (Zinger et al., 2021). A further inheritance analysis of a selected tolerant genotype and the resistant genotype showed that the tolerance trait is controlled by a single recessive gene whereas the resistance trait is controlled by at least two genes (Zinger et al., 2021). One more recent screening of 44 tomato materials by Kabas and colleagues identified four accessions, LA1651 (*S. pimpinellifolium*), LA0716 (*S. penellii*), LA4117 (*S. chilense*), and LA2747 (*S. chilense*), that are tolerant to ToBRFV (Kabas et al., 2022). Although characterization of genetics underlying these identified resistance/tolerance sources and further introgression of the resistance/tolerance genes from wild germplasms into cultivated tomatoes are technically challenging and time‐consuming, this work holds great promise to control ToBRFV.

Genetic transformation is a well‐established technology that has been extensively used to engineer genetic resistance into elite cultivars against devastating viruses in a relatively short time. Transgenic plants expressing the genes or sequences of a pathogen can provide resistance to the same or related pathogens, which is termed pathogen‐derived resistance (PDR). PDR is mediated by RNA silencing (also RNA interference, RNAi), a conserved defence mechanism triggered by double‐stranded RNA (dsRNA) in eukaryotes and the primary antiviral response in plants (Guo et al., 2019; Li & Wang, 2019). The Beachy group first demonstrated that transgenic tobacco plants expressing the CP of TMV was partially resistant to TMV (Abel et al., 1986). Antiviral resistance (also the gene silencing effect) was significantly improved by using a transgene expression cassette to generate an intron‐spliced, inverted repeat RNA sequence, which forms a hairpin RNA or dsRNA on splicing (Smith et al., 2000). The transgene cassette may be further improved to simultaneously target multiple viruses. For instance, transgenic potato plants expressing fused viral CP‐coding sequences from *Potato virus X*, *Potato virus Y*, and *Potato virus S* as a 600‐bp inverted repeat showed nearly 100% resistance to these three viruses (Hameed et al., 2017). In recent years, genetic transformation has also been used for the development of antiviral resistance through transgene‐expressed artificial microRNA (amiRNA) targeting specific viruses or, in combination with a CRISPR/Cas system, by the expression of a single‐guide RNA targeting a specific virus (Chandrasekaran et al., 2016; Mahas et al., 2019; Miao et al., 2021; Zhang et al., 2018). As microRNA (miRNA) plays a pivotal role in RNAi‐mediated defence, it is also possible to engineer genetic resistance through manipulation of tomato‐encoded miRNAs to directly target the specific loci of the ToBRFV genome (Gaafar & Ziebell, 2020). In addition, genetic transformation‐mediated introduction of *R* genes is another elegant solution to a number of viruses, including tobamoviruses (Tamborski & Krasileva, 2020). In cases where the *R*‐mediated resistance has been overcome, random mutagenesis as well as stepwise artificial evolution may be employed to generate *R* mutants to restore resistance (Tamborski & Krasileva, 2020). For instance, *Sw‐5b* confers strong resistance to TSWV and new isolates with two single mutations C118Y or T120N in the NSm protein can overcome *Sw‐5b*‐mediated immunity (Huang et al., 2022). Using a stepwise artificial evolution strategy, Huang and colleagues successfully generated two *Sw‐5b* mutants that confer resistance to TSWV in transgenic plants (Huang et al., 2021). All the approaches discussed here can be adapted to engineer genetic resistance to ToBRFV. However, as long as genetic transformation is involved, public concerns about genetically modified organisms still remain as a big hurdle to pass before the commercialization of any possible tomato cultivars with engineered resistance to ToBRFV.

Plant viruses have a small genome with limited coding capacity and thus rely on a variety of host factors, also known as susceptibility factors, to establish successful infection (Wang, 2015). Thus, mutation or silencing of a host factor gene can lead to inheritable recessive genetic resistance to viruses (Hashimoto et al., 2016; Truniger & Aranda, 2009; Wang, 2015). The well‐characterized recessive resistance genes *eIF4E*/*4G* and their isoform mutants have been validated to be efficient against some plant viruses, particularly potyviruses (Tang et al., 2020; Wang & Krishnaswamy, 2012). Once host factor genes are identified, the precise genome‐editing technology may be employed to mutate or silence them via genetic transformation, followed by removal of the transgene via traditional breeding to obtain transgene‐free “green mutants” (Wang, 2015). *TOBAMOVIRUS MULTIPLICATION1* (*TOM1*) encodes a seven‐pass transmembrane protein that interacts with tobamoviral replication proteins and is indispensable for efficient multiplication of tobamoviruses (Yamanaka et al., 2000, 2002). Simultaneous mutations of *TOM1* and its putative paralog *TOM3* restrict tobamovirus infection in *Arabidopsis* (Yamanaka et al., 2002). Tomato encodes five *TOM1* homologous genes. Recently, this group generated tomato quadruple *tom1* mutants with CRISPR/Cas9 technology and found that the quadruple‐mutant plants grew and developed as wild‐type plants but were highly resistant to four tobamoviruses, including ToBRFV (Ishikawa et al., 2022). This exciting work represents a breakthrough in the development of genetic resistance to ToBRFV through advanced biotechnology. An alternative approach for the development of recessive resistance is to generate mutagenized populations induced by chemical and physical mutagens, such as ethylmethane sulfonate (EMS) or gamma‐rays, and then screen for target host factor mutants using TILLING or other genomics tools (Yang et al., 2021). This approach was successfully used previously to obtain *eif4e* mutants with resistance to two potyviruses in tomato (Piron et al., 2010). If no host factors are available, one may directly screen mutant populations for novel recessive resistance by infection assay. The availability of powerful high‐throughout sequencing and metagenomics tools can accelerate the molecular characterization of the resistance lines identified and the genetics associated with the novel resistance to facilitate breeding for ToBRFV‐resistant cultivars.

In addition to the above control strategies, other novel approaches are also under investigation. For example, Iobbi and co‐workers reported that autoxidation products of the methanolic extract from *Combretum micranthum* leaves, 4‐hydroxybenzoic acid (the main product of the alkaline autoxidation of the methanolic extract) and catechinic acid (a common product of rearrangement of catechins in a hot alkaline solution), have anti‐ToBRFV activity (Iobbi et al., 2022). Molecular docking simulation suggests that 4‐hydroxybenzoic acid and catechinic acid target the amino acid residues responsible for ToBRFV CP–CP interactions, which may affect CP structural stability (Iobbi et al., 2022). This work raises the possibility of using natural plant compounds against ToBRFV.

## 6 CONCLUSIONS AND FUTURE PROSPECTS

Plant viruses are a major constraint to agriculture, accounting for nearly 50% of newly emerging plant diseases and causing an estimated economic loss greater than $30 billion annually (Yang et al., 2021). ToBRFV is a newly discovered, highly contagious, and destructive tobamovirus with tomato as the primary host. Within just a few years of its discovery in Israel and Jordan, the virus has spread to a number of countries across the world. In the past 3 years, a lot of research effort has been devoted to the understanding of ToBRFV transmission modes, distribution, host range, prevention measures, and the development of diverse specific and time‐saving detection methods. Exciting research has also been carried out to screen for resistant germplasms and explore resistance‐breaking mechanisms and the molecular plant–ToBRFV interaction. Tobamoviruses such as ToBRFV and TMV are seedborne and highly contagious through various mechanical contact means and their virions are extremely stable. Therefore, it is not surprising that common prevention measures such as elimination of infected plants and debris, disinfection of seeds and contaminated tools and greenhouses, and crop rotation may slow down but cannot effectively stop the ToBRFV endemic. Growers, breeders, and researchers should be well prepared for a long‐term protracted battle against ToBRFV. The following are some key research areas that are strongly recommended as immediate research priorities:

- The development of attenuated ToBRFV variants to control ToBRFV through cross‐protection to provide a fast and effective solution in the short term.
- Screening for resistance in tomato germplasms and subsequent introgression of identified resistance genes into elite cultivars. Although this approach usually takes a very long time to succeed, positive outcomes of durable resistance will benefit the industry more profoundly.
- Induction of mutagenized tomato populations and screening for novel resistance. The advanced technologies available may accelerate the integration of identified resistance into elite cultivars through breeding programmes.
- New control strategies such as host factor‐based resistance. Research is needed to better understand the ToBRFV infection process and the molecular plant–ToBRFV interaction. The search for ToBRFV host factors required for ToBRFV multiplication and research on mechanics under breakdown and counterbreakdown of *Tm‐2* ^*2*^‐mediated durable resistance by tobamoviruses are of particular interest.
- Impact of ToBRFV on pepper production. So far, most ToBRFV research has been done in tomato and very little has been done in pepper. Differential responses of pepper plants harbouring different *L* alleles to ToBRFV inoculation/infection require further evaluation. The mechanisms underlying these responses are to be investigated.

## CONFLICT OF INTEREST

The authors declare that no competing interests exist.

## Data Availability

Data sharing is not applicable to this article as no new data were created or analysed.
